# A Streamlined Protocol for Wheat (*Triticum aestivum*) Protoplast Isolation and Transformation With CRISPR-Cas Ribonucleoprotein Complexes

**DOI:** 10.3389/fpls.2020.00769

**Published:** 2020-06-10

**Authors:** Kali M. Brandt, Hilary Gunn, Nathalia Moretti, Robert S. Zemetra

**Affiliations:** Wheat Breeding and Genetics, Crop and Soil Science, Oregon State University, Corvallis, OR, United States

**Keywords:** wheat, protoplast, transformation, CRISPR, GFP, ribonucleoprotein, sgRNA

## Abstract

The genetic engineering method CRISPR has been touted as an efficient, inexpensive, easily used, and targeted genetic modification technology that is widely suggested as having the potential to solve many of the problems facing agriculture now and in the future. Like all new technologies, however, it is not without challenges. One of the most difficult challenges to anticipate and detect is gene targets that are inaccessible due to the chromatin state at their specific location. There is currently no way to predict this during the process of designing a sgRNA target, and the only way to detect this issue before spending time and resources on full transformations is to test the cleavage ability of the sgRNA *in vivo*. In wheat, this is possible using protoplast isolation and PEG transformation with Cas9 ribonucleoprotein complexes. Therefore, we have developed a streamlined protocol for testing the accessibility of sgRNA targets in wheat. The first steps involve digesting wheat leaf tissue in an enzymatic solution and then isolating viable protoplasts using filters and a sucrose gradient. The protoplasts are then transformed using Cas9 ribonucleoprotein complexes via PEG-mediated transformation. DNA is isolated from the CRISPR-Cas-edited protoplasts and PCR is performed to amplify the gene target region. The PCR product is then used to assess the editing efficiency of the chosen sgRNA using Sanger sequencing. This simplified protocol for the isolation and transformation of wheat protoplast cells using Cas9 ribonucleoprotein complexes streamlines CRISPR transformation projects by allowing for a fast and easy test of sgRNA accessibility *in vivo*.

## Introduction

Wheat is one of the staple food crops in the world, and currently feeds more than a quarter of the global population (Bushuk, 1997; Gustafson et al., 2009). Multiple public and private entities work toward improving wheat for each growing region. Currently, no transgenic wheat varieties have been deregulated by any government, and therefore the majority of breeders rely heavily on traditional breeding to improve traits and release new varieties (Stokstad, 2004; Cowan, 2014). However, the process from first cross to varietal release can easily take 10 years or more (Wang et al., 2015). Thus, many researchers are turning to genetic transformation via targeted mutagenesis as a potential alternative to traditional breeding (Bhowmik et al., 2018; Haque et al., 2018; Wang et al., 2018b).

Gene editing is the process of making specific modifications to a known DNA sequence within a cell. These modifications can consist of insertions, deletions, or changes in the gene sequence that cause a desired change in the produced protein (Li et al., 2014; Malzahn et al., 2017). CRISPR (Clustered Regularly Interspaced Short Palindromic Repeats) is a relatively new technique that facilitates gene editing while increasing the efficiency of editing for model and non-model plant species, and has quickly replaced most uses of other gene editing methods (Scheben et al., 2017; Jaganathan et al., 2018). CRISPR-Cas has recently been engineered to have the ability to target any desired sequence in eukaryotic cells, was first used to transform crop plants in 2013, and has since been used to genetically edit multiple crop species (Feng et al., 2013; Li et al., 2013; Nekrasov et al., 2013; Shan et al., 2013; Xie and Yang, 2013; Bortesi and Fischer, 2015).

Transgenic wheat has not been deregulated by any world government, and the societal distrust of genetic engineering technologies is at an all-time high. While much research has been published on CRISPR-Cas9 mediated editing of wheat using plasmid expression vectors, the transformation of wheat using CRISPR-Cas ribonucleoproteins (RNPs) is not common. This is likely because RNPs are less stable and less efficient. Plasmids are more efficient, but integrate into the genome of the target organism, and must be removed with subsequent crossing to unmodified plants. The stigma and regulation of transgenic crops means the use of plasmids for CRISPR transformation could result in future issues for deregulation, varietal releases, and shipping overseas. Conversely, RNPs do not integrate into the genome, are only active for a short time in the target organism before being degraded by the cell’s machinery, and have not shown any integration in previous studies (Woo et al., 2015; Andersson et al., 2018). Therefore, RNP-edited crop plants have the potential to avoid current “genetically modified” regulation in multiple countries and to be more widely accepted by the public. Thus, RNP-mediated editing is an important tool in the future of wheat varietal development.

While CRISPR has been touted as having the ability to target any unique sequence in a genome, this comes with a caveat of its own. There are some regions of the genome that may be inaccessible, most likely due to chromatin being tightly packed around histones in that location (Ondřej et al., 2009; Shan et al., 2014). If the RNP complex cannot reach the intended gene target, no sequence modification will occur. Targeting an inaccessible gene in a whole plant, embryo, callus, or single cell will appear the same as a failed experiment. It is virtually impossible to know if some part of the process failed or if the gene target is inaccessible. Therefore, it is critical to test every potential sgRNA target *in vivo* at the single cell level before attempting to transform an entire organism. It is important to note, however, that the chromatin state of genes is not static and may change over a cell’s or tissue’s life span and may also differ from tissue to tissue, and this should be kept in mind when performing an *in vivo* test (Ondřej et al., 2009).

**FIGURE 1:**
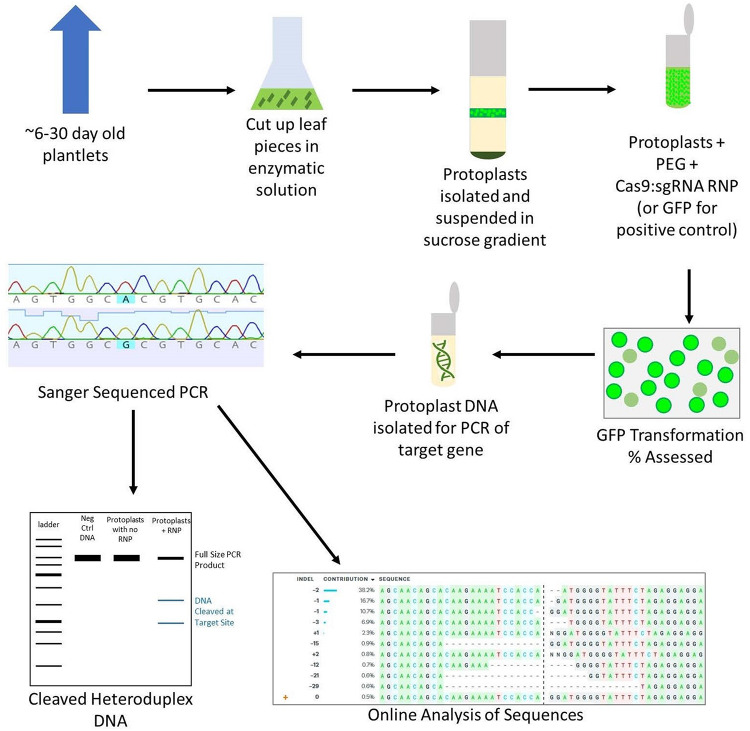
Overview of the protoplast isolation and transformation protocol. Leaf tissue is cut into 2 mm pieces and digested in an enzymatic solution on a shaker. The protoplasts are then filtered and isolated using a sucrose gradient. A PEG-mediated transformation is performed in microcentrifuge tubes, using GFP as a positive control. After 24–48 h, the transformation efficiency is assessed by counting GFP fluorescing cells in the positive control. DNA is isolated from CRISPR-edited protoplasts and PCR is performed. The PCR is then used in a T7EI digestion assay as well as Sanger sequenced and analyzed using an online program to assess the editing efficiency of the chosen sgRNA.

While protoplast regeneration is not currently practical in wheat, protoplasts are the best tool to test transformation *in vivo* and avoid wasting time and resources on failed sgRNA targets (Shan et al., 2014; Wang et al., 2016; Liang et al., 2018). Although many protoplast isolation methods have been reported, there are only two published studies (by the same group) using RNPs to transform wheat protoplasts (Liang et al., 2017, 2018). Other studies have been published using CRISPR to transform wheat protoplasts, but these use plasmid expression vectors rather than RNPs, which adds unnecessary steps and expenses to labs aiming to use RNPs for transformation (Shan et al., 2014; Wang et al., 2016; Cui et al., 2019). In response to the dearth of available protoplast isolation and RNP transformation protocols, we have developed a simplified protocol that requires no specialized equipment, results in high viable protoplast yields, and gives comparable editing percentages to other protocols, including those that use plasmids (Figure 1).

This protocol was tested on two varieties of hexaploid spring wheat: Bobwhite and Chinese Spring, and three gene targets: *GW2-B*, *PinB-D*, and *ASN2-A*. *GW2* is a negative regulator of grain width and weight, and was chosen as a positive control target as it has been used in CRISPR transformation of wheat in multiple published studies. *PinB* is a grain hardness gene that was suspected to be inaccessible to RNPs due to a lack of positive results after many transformation attempts in immature embryos (data not published). *ASN2* is an asparagine synthetase gene in the acrylamide formation pathway that was chosen for its potential as an important breeding target. Each gene target was tested in both varieties a minimum of three times.

## Materials and Equipment

1. Wheat seed: *Triticum aestivum* L. cv. Bobwhite and *Triticum aestivum* L. Chinese Spring.
2. Sunshine^®^ Mix #4 professional growing mix (Sun Gro Horticulture, Agawam, MA, United States).
3. Oligonucleotides for *GW2-B, PinB-D*, and *ASN2-A* (Table 1).

**TABLE 1:** Sequence of oligonucleotides (sgRNA and primers) used to form RNPs and to amplify the *GW2-B, PinB-D*, and *ASN2-A* genes.

**sgRNA name**	**Sequence (5′–3′) (PAM)**		
GW2-B	CCAGGATGGGGTATTTCTAG(AGG)*		
PinB-D	GAAGGGCGGCTGTGAGCATG(AGG)*		
ASN2-A	CCGTCATTTCGCTGGGACGA(AGG)*		

**Primer name**	**Sequence (5′–3′)**	**Tm °C**	**Distance from sgRNA cut site (base pairs)**
GW2-B-F	CTGCCATTACTTTGTATTTTTGTACTC	62	500
GW2-B-R	TCCTTCCTCTCTTACCACTTCCC		220
PinB-D-F	TACTCAGAAGTTGGCGGCTG	62	176
PinB-D-R	GCCCATGTTGCACTTTGAGG		175
ASN2-A-F	GGTCACAAACTATACGCACC	57	185
ASN2-A-R	ACCCTCCGAAGATCTCATC		460

The synthetic sgRNAs were ordered from Synthego (Menlo Park, CA, United States). The primers were ordered from Eurofins Genomics (Louisville, KY, United States). *Be sure to include any necessary nucleotides if using a sgRNA *In Vitro* Transcription Kit.

4. Pipettes and pipette tips (2. 5-, 10-, 200-, and 1000 μL).
5. PCR thermocycler (Eppendorf, Hauppauge, NY, United States).
6. Microcentrifuge tubes (1.5 ml and 2.0 ml) (Eppendorf).
7. DNeasy Plant Mini Kit (Qiagen, Germantown, MD, United States, cat. no. 69104).
8. Analog Vortex Mixer (VWR, Radnor, PA, United States).
9. Benchtop Centrifuge (Eppendorf).
10. NanoDrop spectrophotometer (Thermo Fisher Scientific, Waltham, MA, United States).
11. EnGen^TM^ Spy Cas9 NLS and 10x Cas9 Nuclease Reaction Buffer (New England BioLabs, Ipswich, MA, United States, cat. no. M0646M).
12. Agarose (Genesee Scientific, San Diego, CA, United States, cat. no. 20-102GP).
13. Generuler 1 kb Plus ladder (Thermo Fisher Scientific, cat. no. SM1333).
14. TBE Buffer (Thermo Fisher Scientific, cat. no. B52).
15. Gel *trans*-illuminator system.
16. Analytic balance (Ohaus, Parsippany, NJ, United States).
17. Enzyme Solution (Table 2).

**TABLE 2:** Ingredients for the preparation of Enzyme Solution.

**Chemical**	**Manufacturer**	**Catalog number**	**Quantities**	**Notes on preparation**
MES, free acid	GoldBio, St. Louis, MO, United States	M-095-100	20 mM	Add Type I water to desired volume, pH with NaOH, filter sterilize or autoclave
D-Mannitol	Millipore Sigma, St. Louis, MO, United States	M1902	0.4 M	
KCl	Millipore Sigma	1.04935	10 mM	
NaOH	Fisher Scientific, Waltham, MA, United States	S318-100	pH to between 5.7 and 6.0	
Cellulase R-10	Duchefa Biochemie, Haarlem, Netherlands	C8001	1% (wt/vol)	Shortly before using add cellulase and macerozyme, and warm to 55°C for 10 min
Macerozyme R-10	Duchefa Biochemie	M8002	0.25% (wt/vol)	
CaCl_2_	Millipore Sigma	C8106	10 mM	Cool to room temperature, add CaCl_2_ and BSA
Bovine serum albumin	Millipore Sigma	A2153	0.1% (wt/vol)

18. Glass beakers (140-, 300-, and 1000 mL) (Pyrex).
19. Hot plate stirrer (VWR).
20. Steel scissors.
21. Glass flask (250 mL) (Pyrex).
22. Incubator shaker (Marshall Scientific, Hampton, NH, United States).
23. Mesh snap ball tea strainer.
24. Aluminum foil.
25. W5 Solution (Table 3).

**TABLE 3:** Ingredients for the preparation of W5 solution.

**Chemical**	**Manufacturer**	**Catalog number**	**Quantities**	**Notes on preparation**
MES, free acid	GoldBio	M-095-100	2 mM	Add Type I water to desired volume, pH with NaOH, autoclave or filter sterilize
NaCl	Fisher Scientific	S271-500	154 mM	
CaCl_2_	Millipore Sigma	C8106	125 mM	
KCl	Millipore Sigma	1.04935	5 mM	
NaOH	Fisher Scientific	S318-100	pH to between 5.7 and 6.0

26. W5A Solution (Table 4).

**TABLE 4:** Ingredients for the preparation of W5A solution.

**Chemical**	**Manufacturer**	**Catalog number**	**Quantities**	**Notes on preparation**
Glucose	Millipore Sigma	G8270	5 mM	Add Type I water to desired volume, pH with NaOH, autoclave or filter sterilize
NaCl	Fisher Scientific	S271-500	154 mM	
CaCl_2_	Millipore Sigma	C8106	125 mM	
KCl	Millipore Sigma	1.04935	5 mM	
MES, free acid	GoldBio	M-095-100	0.1% (wt/vol)	
NaOH	Fisher Scientific	S318-100	pH to between 5.7 and 6.0

27. Sucrose (Fisher Scientific, cat. no. S5-500).
28. WI solution (Table 5).

**TABLE 5:** Ingredients for the preparation of WI solution.

**Chemical**	**Manufacturer**	**Catalog number**	**Quantities**	**Notes on preparation**
D-Mannitol	Millipore Sigma	M1902	0.5 M	Add Type I water to desired volume, pH with NaOH, autoclave or filter sterilize
KCl	Millipore Sigma	1.04935	20 mM	
MES, free acid	GoldBio	M-095-100	4 mM	
NaOH	Fisher Scientific	S318-100	pH to between 5.7 and 6.0

29. Pipet filler and serological pipets (10 mL and 25 mL) (Fisher Scientific).
30. Nylon cell strainer (100 μm) (VWR, cat. no. 76327-102).
31. Falcon conical centrifuge tubes (50 mL) (Fisher Scientific).
32. Nalgene^TM^ polysulfone centrifuge tubes (50 mL) (Thermo Fisher Scientific).
33. Evans Blue (Millipore Sigma, cat. no. E2129).
34. GFP reporter plasmid (obtained from Cathleen Ma, Oregon State University, via personal communication).
35. PEG Solution (Table 6).

**TABLE 6:** Ingredients for the preparation of PEG solution.

**Chemical**	**Manufacturer**	**Catalog number**	**Quantities**	**Notes on preparation**
PEG 4000	Millipore Sigma	1546569	40% (wt/vol)	Add Type I water to desired volume, place on a shaker for 1 h or until PEG is dissolved
D-Mannitol	Millipore Sigma	M1902	0.2 M	
CaCl_2_	Millipore Sigma	C8106	0.1 M	

*The solution should be freshly prepared but made at least 1 h before transformation to completely dissolve the PEG.*

36. Hemocytometer.
37. Fluorescence microscope (Leica, Buffalo Grove, IL, United States).
38. Phusion High-Fidelity PCR Master Mix with HF Buffer (Thermo Fisher Scientific, cat. no. F531S).
39. dNTPs (Thermo Fisher Scientific, cat. no. R0182).
40. QIAquick PCR Purification Kit (Qiagen, cat. no. 28104).
41. 10x NEBuffer 2 (New England BioLabs, cat. no. B7003S).
42. T7 Endonuclease I (New England BioLabs, cat. no. M0302S).
43. EDTA (Millipore Sigma, cat. no. 819040).

## Stepwise Procedures

### Cleavage Test Using CRISPR-Cas9 RNPs

Before beginning the *in vivo* transformation steps, a gene target must be selected and an appropriate sgRNA designed and tested *in vitro*. In the case of bread wheat, an allohexaploid, most genes are present in three copies, one for each genome (A, B, and D). Homoeologs may be expressed at different levels from each other and are often compensatory, meaning if one copy is knocked out, expression in another copy will increase to make up for the loss (Slade et al., 2012; Wang et al., 2018a). This is important to keep in mind when deciding which gene and/or copies of that gene to target. For this study, we targeted *GW2-B, PinB-D*, and *ASN2-A*. All three sgRNAs were ordered as synthetic sgRNAs, combined with Cas9 protein, and their functionality tested *in vitro* on RNase-Free DNA from Bobwhite and Chinese Spring.

1. RNase-free DNA is prepared from Bobwhite and Chinese Spring plantlets using the DNeasy Plant Mini Kit and following the manufacturer’s instructions, omitting the addition of RNase A to prevent digestion of the sgRNA transcript.
2. PCR amplification of the gene target is performed using the gene target primers (Table 1) and Phusion High-Fidelity PCR Master Mix following the manufacturer’s instructions.
3. The PCR product is then purified with the QIAquick PCR Purification Kit following the manufacturer’s instructions.
4. The RNPs are formed by mixing 0.1 μl of sgRNA (0.8 μg), 0.5 μl of Cas9 nuclease (1.61 μg), and 2.4 μl of 10x Cas9 Nuclease Reaction Buffer for a total volume of 3 μl RNP per reaction.^∗^
  ^∗^Note: This step should be optimized for the type of sgRNA and Cas9 used. Follow the manufacturer’s instructions accordingly.
5. The RNPs are then incubated at room temperature for 10 min.
6. The RNP complex is added to 200 ng of PCR product, 0.32 μl of 10x BSA, and RNase-Free H_2_O to 30 μl total.
7. The cleavage reaction is incubated at 37°C for 1 to 2 h, followed by 80°C for 5 min.
8. The cleaved PCR product is then run on a 2% agarose gel in 0.5x TBE buffer with a 1 kb Plus ladder. Cleavage activity is assessed based on the amount of digested product compared to the amount of total input DNA.

### Isolation of Protoplasts

Protoplast isolation begins with digesting young leaf tissue in an enzymatic solution to remove the cell walls and free the protoplast cells. The solution is then filtered through a mesh strainer to remove large pieces of tissue, washed to remove small debris, centrifuged in a sucrose gradient to separate dead and viable protoplasts, and finally brought to the desired concentration.

Timing: Growth of plantlets takes up to 3 weeks. Digestion of leaf tissue takes approximately 7 h, and isolation of protoplasts takes approximately 12 h.

1. Plant wheat seeds in autoclaved Sunshine^®^ Mix #4 and grow at 21°C with a 10 h photoperiod for at least 6 days, up to 30 days.
2. Cut 2 g of wheat leaves from young wheat plants and leave to soak in sterile water at 4°C while preparing the solutions.
3. Add enzymes to Enzyme Solution, warm to 55°C for 10 min, cool to room temperature, add CaCl_2_ and BSA.
  ^∗^Note: 2 g of tissue requires 100 ml of Enzyme Solution.
4. Remove wheat leaves from water and cut into 2 mm pieces using scissors, put cut pieces into 0.4 M D-mannitol.
5. Once enzyme solution is ready and all leaf pieces have been cut, use a mesh snap ball tea strainer to remove leaves from mannitol solution.
6. Put leaf pieces into a flask containing the enzyme solution, cover the flask completely with aluminum foil, and place on an incubating shaker at 27°C at 100 rpm for 3 h.
7. Gently swirl the flask to release the protoplasts.
8. Filter the solution through a mesh snap ball tea strainer into a small beaker. Rinse the flask and leaf pieces with 20 ml of W5 solution.
9. Filter the liquid through a 100 μm cell strainer into 50 ml Falcon tubes. Rinse the strainer with 1–2 ml of W5 solution.
10. Distribute evenly into Nalgene^TM^ centrifuge tubes.
11. Centrifuge at 100 × *g* for 5 min.
12. Being very careful not to disturb the pellet, remove the supernatant by pipetting.
13. Resuspend the pellet in 4 ml of W5A solution.
14. Add 8 ml of filter sterilized 21% sucrose solution to a new Nalgene^TM^ centrifuge tube.
15. Very slowly and carefully, layer the protoplast solution on top of the sucrose.
  ^∗^Note: Always cut the ends off of the pipette tips whenever transferring protoplasts in order to prevent shearing them.
16. Centrifuge at 720 × *g* for 13 min.
17. There should be a layer of clear W5A Solution on top, followed by a small layer of viable green protoplasts, then a large layer of sucrose, and then dead/broken protoplasts at the bottom.
18. Slowly harvest the viable protoplasts by pipetting, and place in 2.0 ml microcentrifuge tubes, up to 1 ml of protoplasts per tube.
19. Bring total volume in each tube to 2.0 ml with WI solution.
  ^∗^Note: Without WI solution, the protoplasts may not settle to the bottom, but remain suspended in solution.
20. Cover the tubes with aluminum foil and leave at 4°C overnight to let the protoplasts settle to the bottom.
21. Once settled, pipette off the supernatant.
22. Check protoplast concentration with a hemocytometer.
23. Check protoplast viability with 1% Evans Blue dye by adding 3 μl of dye per 100 μl of protoplasts and incubating for 10 min at room temperature. Any dead tissue will be dyed blue.
24. Make up to desired concentration with WI solution (optimal concentration is 0.7–1.0 × 10^6^ cells/ml).
a. Save some protoplasts at 4°C to use as a negative control in step 1 of Extraction of DNA, T7EI Digestion, and Detection of Mutations.

### PEG-Mediated Transformation

Once the protoplast solution has been made up to the working concentration, it is ready to be used for transformation. The RNPs are formed first in an RNase-free environment, then they are combined with freshly made PEG and the protoplasts. The transformation is stopped with WI solution and then the mixture is washed to remove PEG, before being placed at room temperature in the dark for 48 h to allow the RNPs to edit the cells.

Timing: PEG-mediated transformation takes 1 to 2 days.

1. The RNPs are formed by mixing 2 μl of sgRNA (16 μg), 10 μl of Cas9 nuclease (32.2 μg), and 8 μl of 10x Cas9 Nuclease Reaction Buffer for a total volume of 20 μl RNP per reaction, and incubated for 10 min as room temperature.
  ^∗^Note: This step should be optimized for the type of sgRNA and Cas9 used. Follow the manufacturer’s instructions accordingly.
2. Add RNPs to 50 μl of protoplasts (at 0.7–1.0 × 10^6^ cells/ml).
  ^∗^Note: The volumes of protoplasts and RNPs can be increased if more DNA is required from the DNA extraction.
3. Immediately add the same volume in μl of freshly prepared PEG solution as that of the RNP and protoplast solution (so that the final volume is approximately 50% PEG solution), and mix thoroughly by gently inverting the tube until homogenous.
  ^∗^Note: DNA, protoplasts, and PEG solution should be added to the microcentrifuge tube in that order. Don’t delay in between adding protoplasts and PEG. Once protoplasts and DNA have been combined, add PEG immediately.
4. **For the positive control:** Add 15 μg of GFP reporter plasmid to protoplasts instead of RNPs (15 ug of GFP plasmid in 40 μl or less + 50 μl of protoplasts at 0.7–1 × 10^6^ cells/ml), followed by PEG solution as in step 3 above.
5. Incubate mixture for 15–20 min at room temperature in the dark. Add two times the volume of WI solution to the tube (e.g., add 800 μl WI solution to 400 μl of RNPs + protoplasts + PEG) and mix well by inverting the tube to stop the transformation process.
6. Centrifuge the tubes at 150 × *g* for 3 min at room temperature. Remove the supernatant by pipetting.
7. Resuspend protoplasts gently to 1 ml with WI solution.
8. Coat 2.0 ml microcentrifuge tubes with 5% BSA (5 mg BSA in 1 ml sterile water) to prevent protoplasts from sticking to the plastic and each other. Add enough BSA to coat all surfaces then pour it out. The BSA can be re-used for multiple tubes.
9. Immediately transfer all of the protoplast solution into the wet BSA-coated tubes.
10. Wrap the tubes with aluminum foil, lay them on their sides, and incubate at 23°C for 24 to 48 h.
  a. GFP expression peaks after 24 h if using a transient plasmid.
  b. RNPs should be allowed to incubate for 48 h.
  c. Check the transformation efficiency by comparing the number of GFP-fluorescing cells to non-fluorescing cells in the positive control using a fluorescent microscope and hemocytometer.
11. Collect the protoplasts by centrifuging at 12,000 × *g* for 2 min at room temperature. Remove the supernatant by pipetting.
  ^∗^Note: Remember to also collect the protoplasts from the negative control sample from step 24a of Isolation of Protoplasts.

### Extraction of DNA, T7EI Digestion, and Detection of Mutations

After incubation with the RNPs, DNA is extracted from the protoplasts and the target sequence is amplified via PCR. The PCR product can then be tested for editing using a T7EI digestion and Sanger sequencing followed by analysis with a program such as TIDE (Tracking of Indels by Decomposition) (Brinkman et al., 2014) or ICE (Inference of CRISPR Edits) (Hsiau et al., 2019). TIDE and ICE are bioinformatics software tools developed to analyze pooled CRISPR-edited DNA. They use the sequence traces from Sanger sequencing to reconstruct all indels, their frequencies, and quality control metrics, thus providing detailed information about the types and sizes of indels, as well as specific nucleotide changes. We recommend using these programs instead of a T7EI digestion, as they are more accurate and more informative. After confirmation of the sgRNA functionality *in vivo*, it can be used for editing a whole organism using any method.

Timing: The extraction of protoplast DNA takes approximately 4 h. PCR of the extracted DNA and heteroduplex formation takes approximately 5 h. The T7EI digestion takes approximately 2 h.

1. Extract genomic DNA using the DNeasy Plant Mini Kit.
2. Determine the DNA concentration with a Nanodrop spectrophotometer. The usual concentration is around 30 ng/μl in a total volume of 30 μl.
  ^∗^Pause point: Extracted DNA can be stored at −20°C for several months, or 4°C for several weeks.
3. Set up the PCR reaction to amplify the genomic region targeted for mutagenesis (Table 7).

**TABLE 7:** PCR reaction for amplifying the desired genomic region.

**Component**	**Amount (μl)**	**Final concentration**
Phusion HF buffer, 5x	5	1x
dNTP, 2 mM	2.5	0.2 mM
PCR-Fwd primer, 10 μM	1.25	0.5 μM
PCR-Rev primer, 10 μM	1.25	0.5 μM
Phusion HF polymerase, 2 U/μl	0.25	0.5 units
DNA template	8	3–6 ng/μl
ddH_2_O	To 25	

^∗^It is important to use high-fidelity polymerase to reduce the error rate, and to use the untransformed protoplast DNA from step 11 of PEG-Mediated Transformation as a negative control.

4. Perform the PCR for amplifying the desired genomic region (Table 8).

**TABLE 8:** PCR reaction parameters.

**Step number**	**Denature**	**Anneal**	**Extend**
1	98°C, 30 s		
2–4	98°C, 10 s	62°C, 30 s, −0.5°C per cycle	72°C, 30 s
5	Go to 2, 8 times		
6–8	98°C, 10 s	58°C, 30 s	72°C, 30 s
9	Go to 6, 32 times		
10			72°C, 5 min

*The PCR conditions may change depending on the primers and type of polymerase used.*

5. Clean up the PCR with a kit of your choice following the manufacturer’s instructions.
  a. At this point, perform Sanger sequencing of each sample and use CRISPR analysis software to quantify the editing rate and determine the type of change.
  ^∗^Pause point: PCR products can be stored at 4°C for several weeks.
6. For the T7EI digestion, set up an annealing reaction to form DNA heteroduplexes by adding 200 ng PCR product, 2 μl 10X NEBuffer 2, and nuclease-free water up to 19 μl total.
7. Run the annealing reaction in a thermocycler using the following conditions: 95°C for 5 min, 95–85°C (−2°C/s), 85–25°C (−0.1°C/s), 4°C forever.
8. Set up the T7EI nuclease digestion by adding 1 μl of T7EI to 19 μl of heteroduplexed DNA.
9. Mix well and spin the mixture down briefly. Incubate the reaction at 37°C for 15 min, then stop the reaction with 1.5 μl of 0.25 M EDTA.
  a. The incubation time can be increased if necessary.
10. Run digestion products on a 2% agarose gel in 0.5x TBE buffer using standard protocols. Include a DNA ladder and negative controls on the same gel. If digested bands are only observed in the CRISPR-Cas9-treated sample, and not in the negative control, an indel mutation has occurred.

### Potential Experimental Pitfalls

1. Low protoplast concentration and/or viability after isolation. This may be due to varietal differences and will require some adjustments to the concentration of enzymes in the enzyme solution and/or the length of digestion. It may also be due to the tissue age, as levels of cellulose and lignin increase over time, potentially inhibiting enzymatic digestion. To prevent this, be sure to use leaf tissue that is less than 4 weeks old.
2. Low transformation percentages of GFP plasmid control. Ensure that the PEG solution is freshly prepared and that it has completely dissolved before using. Also be sure that your ratios of DNA to protoplasts are within the recommended range, as a slightly low concentration of GFP plasmid can significantly reduce transformation efficiencies.
3. Low concentration of protoplast DNA after extraction. Ensure the protoplasts being transformed are viable. The sucrose gradient separation step, refrigeration overnight, and storage in WI solution should maintain viable protoplasts. If the problem persists and protoplasts are viable, increase the overall number of protoplasts used while keeping the ratios within acceptable ranges.

### Application and Limitations

While this protocol was specifically adapted for the assessment of RNP transformation in wheat leaf protoplast cells, there is the potential for it to be applied to other plant species and CRISPR delivery methods, with some modifications. The protoplast isolation steps can also be followed for use with different applications in addition to transformation with PEG and RNPs.

## Results and Discussion

### Protoplast Isolation

**FIGURE 2:**
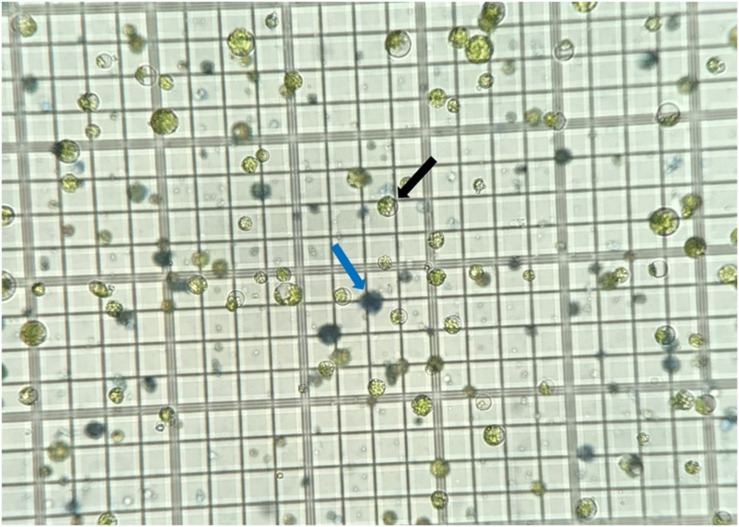
Protoplasts stained with Evans Blue dye on a hemocytometer. Viable cells are bright green and indicated by the black arrow. Nonviable, dead cells are blue and indicated by the blue arrow.

The protoplast isolation procedures described above were repeated at least six times for both Bobwhite and Chinese Spring. Two grams of leaf tissue in 100 ml of enzymatic solution consistently gave total protoplast yields averaging 1.8 × 10^6^ and 1.2 × 10^6^ viable cells at step 22 of Isolation of Protoplasts for Bobwhite and Chinese Spring, respectively. Yields of this amount were easily brought to a concentration of 0.7 to 1.0 × 10^6^ cells/ml for use in transformation. On average, Evans Blue assays showed 16% and 9% dead protoplasts for Bobwhite and Chinese Spring, respectively, at this stage (Figure 2). The protoplasts survived storage in 2.0 ml microcentrifuge tubes in the dark at 4°C for up to 3 days after isolation and concentration at step 24 of Isolation of Protoplasts before viability decreased to below 0.7 × 10^6^ cells/ml.

Protoplasts from the varieties Bobwhite, Kenong199, and Fielder have successfully been transformed with CRISPR plasmids (Shan et al., 2014; Wang et al., 2016; Cui et al., 2019); but only Kenong199 and YZ814 have been transformed with CRISPR RNPs (Liang et al., 2017, 2018). Bobwhite and Chinese Spring were used in this study, as they are commonly found in the United States and are therefore easy for researchers in this region to obtain. We were able to utilize the same protocol for both cultivars and obtain protoplast yields well above 1.0 × 10^6^ cells/ml.

Shan et al. (2014), Wang et al. (2016), Liang et al. (2017, 2018), and Cui et al. (2019) do not report the viability of protoplasts after isolation. It is important for researchers using protoplast isolation protocols to obtain healthy, viable cells without a large number of nonviable cells so that the editing rates are detectable, as a large number of nonviable cells can result in false negative edits. The addition of the sucrose gradient separation at step 17 of Isolation of Protoplasts removes many of the broken nonviable protoplasts from the final mixture and can help to increase transformation efficiency, especially if the number of nonviable cells is high after digestion.

In the other CRISPR protoplast transformation studies mentioned above, vacuum infiltration was used before enzymatic digestion, and is considered to be a “critical step for good protoplast yield” (Shan et al., 2014). Wang et al. (2016) and Cui et al. (2019) reported final isolation concentrations of 2.5 × 10^5^ cells/ml and 1 × 10^6^ cells/ml, respectively, though neither reported final volumes or number of viable protoplasts. The studies by Shan et al. (2014), Liang et al. (2017, 2018) for the protoplast isolation procedure, as all three reports are from the same research group. Therefore, the only results for these three studies are in Shan et al. (2014) which reports yields of 1 × 10^7^ cells per 50 ml digestion with a final concentration of 2.5 × 10^6^ cells/ml. In this study, vacuum infiltration was omitted, and the resulting protoplast yield of over 1 × 10^6^ cells per digestion is comparable to or even exceeds those of the other protocols using vacuum infiltration (Shan et al., 2014; Wang et al., 2016; Liang et al., 2017, 2018; Cui et al., 2019). This protocol offers researchers a more comprehensive result using simplified methods with less specialized equipment by reporting cell viability, protoplast yields, final concentration, and omitting vacuum infiltration altogether.

### PEG-Mediated Transformation

The PEG-mediated transformation of Bobwhite and Chinese Spring protoplasts was repeated at least three times for each gene target in each cultivar. The most consistent, best performing ratios in this study were between 0.0004 and 0.002 μg of RNPs to total protoplast number, and 0.2 to 0.4 μl of RNPs to total μl protoplasts. For the positive control, the best performing ratios were between 0.0002 to 0.0004 μg of GFP plasmid to total protoplast number, and 0.4 to 0.6 μl of GFP plasmid to total μl protoplasts (Table 9). A low concentration of sgRNA and/or Cas9 will lead to a high volume of RNPs needed for editing, which will dilute the mixture to the point where RNPs will not come in sufficient contact with the protoplasts, and lead to false negative or low editing rates. Low protoplast concentrations will lead to the same result due to the large volume of protoplasts required. A high protoplast concentration will also likely lead to false negative or low editing rates due to the number of protoplasts saturating the amount of RNPs in the assay, leaving a large number of cells never incorporating RNPs. Therefore, it is important to stay within the recommended ratios not only for the amounts of protoplasts and RNPs, but also for the volumes of protoplasts and RNPs. These ratios are recommended for researchers first implementing this protocol to provide more flexibility for varying sgRNA concentrations, Cas9:sgRNA binding abilities, protoplast concentrations, and cost of reagents. The reported GFP plasmid ratios also may need to be optimized depending upon the source of GFP and the strength of the promoter used.

**TABLE 9:** Ideal ratios of amount of proteins to number of protoplasts, and volume of proteins to volume of protoplasts for GFP control and for CRISPR RNPs.

**For GFP control**	μg of GFP plasmid/total protoplast number	0.0002 to 0.0004
	μl of GFP plasmid/total μl protoplasts	0.4 to 0.6

**For RNPs**	μg of RNPs/total protoplast number	0.0004 to 0.002
	μl of RNPs/total μl protoplasts	0.2 to 0.4

Shan et al. (2014), Wang et al. (2016), Liang et al. (2017, 2018), and Cui et al. (2019) recommend a single volume and/or concentration for all components of the PEG-mediated transformation rather than a range of acceptable values. The PEG-mediated transformation steps reported in each of the published protocols mentioned above also show deviations in concentration of cells used, volume of cells, final number of cells, volume of RNP or plasmid, and amount of RNP or plasmid (Table 10). These disparate numbers further illustrate the potential for the use of a range of acceptable ratios in PEG-mediated CRISPR transformation of wheat protoplasts that would benefit researchers, since it is sometimes difficult to achieve the exact concentrations and volumes of sgRNA, RNPs, plasmids, and protoplasts recommended by the other studies. A range of acceptable values for editing protoplasts as reported in this study facilitates realistic lab situations and makes implementation of this protocol easier, especially considering the many potential sources, yields, and concentrations of sgRNA and Cas9.

**TABLE 10:** Summary of CRISPR transformation methods in wheat protoplasts available to date, with volumes and amounts of reagents used in each transformation protocol.

**Protocol**	**Cell concentration used (cells/ml)**	**Volume used (μl)**	**Final number of cells used**	Volume of plasmid*or RNP^†^ used (μl)	Amount of plasmid* **or RNP^†^ used (μg)**	Ratio of μg plasmid* **or RNP^†^ to number of protoplasts**	Ratio of μl plasmid* **or RNP^†^ to μl protoplasts**
Shan et al. (2014)	2.5 × 10^6^	200	5 × 10^5^	40*	4*	0.00008*	0.2*
Wang et al. (2016)	1 × 10^6^	100	1 × 10^5^	10–20*	10*	0.0001*	0.1–0.2*
Cui et al. (2019)	2.5 × 10^5^	100	2.5 × 10^4^	10*	10*	0.0004*	0.1*
Liang et al. (2017)	2.5 × 10^6^	200	5 × 10^5^	40^†^	40^†^	0.00008^†^	0.2^†^
Liang et al. (2018)	2.5 × 10^6^	200	5 × 10^5^	20–30^†^	40^†^	0.00008^†^	0.1–0.15^†^
This protocol	1 × 10^6^	50	5 × 10^4^	20^†^	48.2^†^	0.001^†^ (0.0004–0.002)^‡^	0.4^†^ (0.2–0.4)^‡^

*Plasmid. ^†^RNP. ^‡^The reported ratios performed the best for our sgRNAs and Cas9 protein. The ranges in parentheses are ratios that were also successful using this protocol and are recommended for other researchers beginning protoplast transformations.

### GFP Expression

**FIGURE 3:**
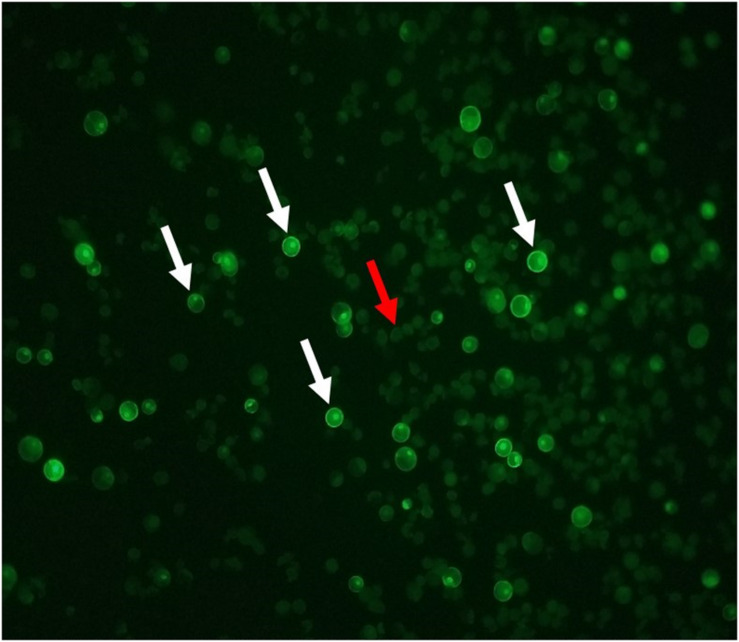
GFP fluorescing protoplast cells. Cells with bright green borders and nuclei are expressing the GFP plasmid. A selection of GFP fluorescing cells are indicated with white arrows. Solid, light green cells are untransformed, as indicated by the red arrow.

A GFP plasmid transformation was performed with every round of RNP transformations as a positive control for the functionality of the PEG. The GFP plasmid transformation rate was consistently above 27% for both Bobwhite and Chinese Spring when using the suggested ratios (Figure 3). Shan et al. (2014) anticipated a GFP plasmid transformation efficiency of 70 to 80%. Cui et al. (2019) reported approximately 60% transformation in the cultivar Roblin, which was not used for the CRISPR transformation steps. Wang et al. (2016) reported approximately 60% transformation efficiency. Liang et al. (2017) did not report GFP plasmid transformation efficiencies, but Liang et al. (2018) anticipated a 50% or greater transformation efficiency. GFP expression plasmids can differ in promoter type, number of promoters, presence and number of enhancer regions, presence and type of nuclear localization signal, and many more features that affect uptake, incorporation, and expression of the gene (Addgene, 2020). Therefore, a specific GFP plasmid transformation efficiency is not recommended for users of this protocol. Instead, it is suggested that researchers begin by using the recommended ratios in Table 9 to obtain at least 20% GFP plasmid transformation efficiency. Then the transformation efficiency of *GW2-B* can be assessed. If it is not at least 30%, the ratios used during transformation can be adjusted until a sufficient efficiency is reached. This will facilitate implementation of this protocol in diverse laboratories with access to various GFP expression plasmids.

### T7EI Digestion

**FIGURE 4:**
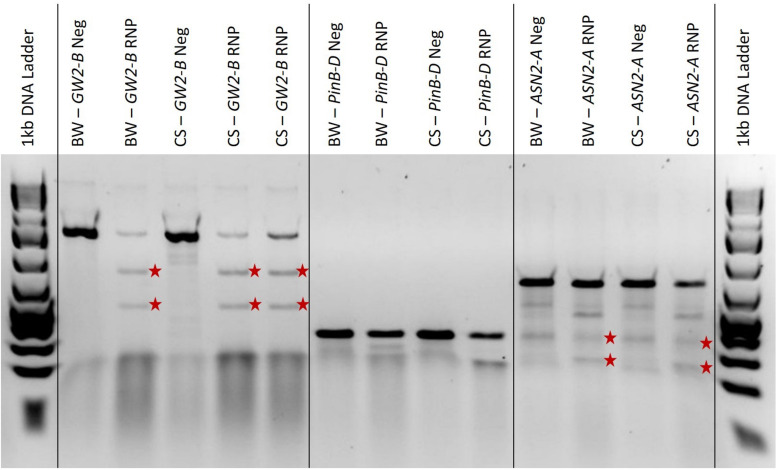
A 2% agarose gel with T7EI digested PCR, negative controls, and a 1 kb Plus ladder on each end. ‘BW’ and ‘CS’ refer to the Bobwhite and Chinese Spring cultivars, respectively. ‘Neg’ means the sample is from protoplasts that were not edited with RNPs and are used as a negative control. ‘RNP’ means that the protoplasts were edited with RNPs targeting the gene indicated. The red stars are to the right of T7EI digested bands of the expected length for each gene target. The amplified *GW2-B* gene target is 1270 bp long, with expected cut fragments of 760 and 510 bp. The amplified *PinB-D* gene target is 351 bp long, with expected cut fragments of 175 and 176 bp. The amplified *ASN2-A* gene target is 645 bp long, with expected cut fragments of 185 and 460 bp.

We found that the T7EI digestion time should be altered to match the expected transformation efficiency based on the GFP transformation percentage. If GFP plasmid transformation is low, around 10%, the digestion time should be increased up to 1 h. If GFP plasmid transformation is high, around 60%, the digestion time can be left at 15 min (data not published). This ensures that even with low transformation rates, the digested bands will become concentrated enough to be visible on the electrophoresis gel. In Figure 4, the three *GW2-B* edited samples showed clear bands at the expected 510 bp and 760 bp sizes; the two *PinB-D* edited samples are not as clear and may have bands around the expected 175 bp and 176 bp sizes, especially in the Chinese Spring sample; and the *ASN2-A* samples all show a bit of extra banding, but the edited samples appear to have bands at both expected sizes of 185 bp and 460 bp. The *GW2-B* gene target shows a clean digestion with T7EI, and is a candidate for determining editing efficiencies using band intensity. *PinB-D* and *ASN2-A*, however, have extra banding in both the edited and negative control samples that may adversely affect the determination of editing efficiencies.

The results shown in Figure 4 are relatively useful for determining whether or not there is editing present in each sample, but the gel image is more difficult to interpret for *PinB-D* and *ASN2-A*, and quantification would be a challenge. It has been shown that the accuracy of the T7EI assay can be affected by the size of the indel, the type of nucleotides added or deleted, secondary structures, flanking sequence, and mutant sequence abundance (Picksley et al., 1990; Vouillot et al., 2015; Sentmanat et al., 2018). A recent study found that T7EI assays underreported CRISPR-Cas9 editing efficiencies by 46% on average. They also found that T7EI assays are especially inaccurate with sgRNAs that have less than a 10% editing efficiency and sgRNAs that have greater than 90% editing efficiency (Sentmanat et al., 2018). Therefore, this assay is only recommended to be used for validation before sequencing, if desired, and not for quantification of editing efficiency.

### Detection of Mutations

Sanger sequencing results are usually given as chromatograms with the occurrence of nucleotides represented as colored peaks. The chromatograms of mutated protoplast DNA do not typically give any discernable indication of the mutation efficiency or type of mutations due to the variation in types of edits in each protoplast cell, and due to the presence of un-mutated protoplast cell DNA in each sample. Therefore, the changes must be visualized using software developed for deconvoluting pooled sequence data from CRISPR transformations such as TIDE or ICE (Brinkman et al., 2014; Sentmanat et al., 2018; Hsiau et al., 2019). Although not as accurate as next generation sequencing (NGS), deconvolution software has been shown to be comparable to NGS for identifying frequencies and identities of indels occurring at 5% or greater.

In this study, all PCR products that showed successful T7EI digestions were sequenced using Sanger sequencing, analyzed with the online program ICE, and total editing frequencies were averaged to obtain the final editing efficiencies. The *GW2-B* sgRNA editing efficiency was 19.2% in Bobwhite and 36% in Chinese Spring, with standard deviations of 20.6% and 22.3%, respectively; the *ASN2-A* sgRNA efficiency was 16.4% in Bobwhite and 12.9% in Chinese Spring, with standard deviations of 12.8% and 10.8%, respectively; and the *PinB-D* sgRNA efficiency in both Bobwhite and Chinese Spring was 0%, with a standard deviation of 0% for both.

**FIGURE 5:**
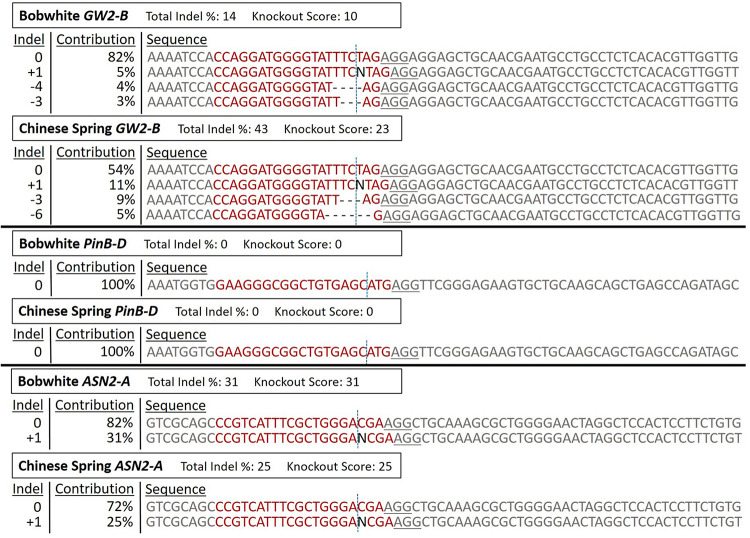
ICE Analysis of CRISPR RNP edited protoplast cells. The RNP edited samples from Figure 4 were sequenced with Sanger sequencing, and then analyzed with the online program ICE to deconvolute the pooled DNA. ‘Bobwhite *GW2-B*’ corresponds to ‘BW-*GW2-B* RNP,’ ‘Chinese Spring *GW2-B*’ corresponds to the first ‘CS-*GW2-B* RNP’ sample, ‘Bobwhite *PinB-D*’ corresponds to ‘BW-*PinB-D* RNP,’ ‘Chinese Spring *PinB-D*’ corresponds to ‘CS-*PinB-D* RNP,’ ‘Bobwhite *ASN2-A*’ corresponds to ‘BW-*ASN2A* RNP,’ and ‘Chinese Spring *ASN2-A*’ corresponds to ‘CS-*ASN2-A* RNP’ from Figure 4. For each of these samples, the corresponding negative control from the T7EI assay was used as the control sequence in ICE. ‘Total Indel %’ is the editing efficiency, or the percent of the total pool of protoplast samples that are not wild-type sequences. ‘Knockout Score’ is the proportion of protoplasts in each sample that either have a frameshift mutation or have an indel of 21 nucleotides or more. The sgRNA target sequence is shown in red. The vertical dotted line represents the expected cut site based on the sgRNA sequence. The underlined sequence in gray is the PAM sequence used by Cas9. ‘Indel’ is the number of nucleotides either inserted or deleted in each sequence type. ‘Contribution’ is the percent of all sequences that have each specific indel type.

A representative sample of protoplast DNA sequencing analysis is shown in Figure 5. The PCR products used for Sanger sequencing and ICE analysis in this figure are the same samples as in Figure 4. The Bobwhite and Chinese Spring *GW2-B* samples showed clear digested bands in the T7EI assay, and were shown by ICE to have 14% and 43% editing efficiency, respectively. Both samples had multiple types of indels at frequencies above 3%. The Bobwhite and Chinese Spring *PinB-D* samples appeared to potentially have some edited cells based on the T7EI assay. The ICE analysis, however, showed that none of the protoplast cells were edited with the *PinB-D* sgRNA, further reinforcing the hypothesis that this gene target is inaccessible *in vivo*. Bobwhite and Chinese Spring *ASN2-A* samples appeared to have successfully digested bands in the T7EI assay, but they were not clear and obvious. ICE analysis of these samples showed an editing efficiency of 31% in Bobwhite and 25% in Chinese Spring. In both cultivars the primary type of edit was a single nucleotide insertion at the expected cut site of the sgRNA. Each of the samples that were successfully edited by the RNPs had mutant sequences which were predicted to significantly impact protein function, confirming the potential for *GW2-B* and *ASN2-A* as targets for other CRISPR projects.

Shan et al. (2014), Wang et al. (2016), and Cui et al. (2019) transformed protoplasts using plasmids rather than RNPs. Shan et al. (2014) used a PCR/restriction enzyme (RE) assay to determine a 45% editing efficiency in TaLOX2. Wang et al. (2016) used NGS to assess the editing efficiency of three different gene targets. *TaGW2* (not the same sgRNA target sequence as this study) showed 2.7% editing efficiency in all three genomes; *TaLpx-1* showed editing efficiencies of 0.3 and 6.1% in the B and D genomes for two different experiments; and *TaMLO* showed an editing efficiency of 4.1% in the A genome. Cui et al. (2019) used high throughput sequencing (HTS) to assess the editing efficiency of three different gene targets using two co-expressed sgRNA for each gene. *TaABCC6* had editing efficiencies between 6.6 and 13.0%; *TansLTP9.4* had editing efficiencies between 0 and 11.9%; and *TaNFXL1* had editing efficiencies between 0 and 42.2%.

Liang et al. (2017, 2018) used PCR/RE assays to determine editing efficiencies of protoplasts using RNPs. Liang et al. (2018) designed sgRNAs for *Lox2* and *CER9*, which resulted in 23.8 and 33.6% editing efficiency, respectively. Liang et al. (2017) used the same *GW2* sgRNA sequence as this study, which resulted in an editing efficiency of 33.4% for *GW2-B*. The *GW2-B* editing efficiencies in this study of 19.2% for Bobwhite and 36% for Chinese Spring are comparable to the results from Liang et al. (2017) in Kenong199. They are also well within the ranges of successful protoplast transformations from the other studies and gene targets, both using plasmids and RNPs. The editing efficiency of *ASN2-A* was lower overall for both cultivars, but still comparable to the other studies.

Wang et al. (2016) and Cui et al. (2019) both utilized high-throughput, whole genome sequencing techniques to determine the editing efficiency of each sgRNA. NGS and HTS techniques are not always a feasible option for every situation, however. The accessibility of the sequencing machinery, data analysis ability (both personnel and computing power), amount of time to obtain results, and cost can all be inhibitory in certain situations (Slatko et al., 2018). For researchers wishing to implement CRISPR transformation, a quick, easy, and cost effective way to screen sgRNAs for functionality and efficiency is important. Thus, NGS and HTS are not recommended for use with this protocol.

Shan et al. (2014) and Liang et al. (2017, 2018) used PCR/RE assays to determine editing efficiencies of their sgRNAs. This can be a useful test for determining presence or absence of editing, and as shown by these studies can be used to quantify editing efficiency. PCR/RE assays cannot, however, differentiate between different types of indels or give the individual frequencies of each indel. It is also not always possible to design each sgRNA around a restriction enzyme site, and the need to do so can further limit the number of possible target sites. In bread wheat, the large number of homoeologous genes and pseudogenes can severely restrict sgRNA design. Adding another level of restriction by using PCR/RE assays is not practical and is therefore not recommended.

While NGS techniques are the most accurate for determining sgRNA editing efficiencies, they are not the most accessible. PCR/RE assays are a faster and less expensive method for determining editing efficiencies, but they are not always practical for sgRNA design and do not give details on types or frequencies of individual indels. Sanger sequencing is much more accessible, less expensive, faster, and easier to use than NGS, and is less restrictive and more detailed than PCR/RE assays. Utilizing Sanger sequencing analysis with a program such as TIDE or ICE to determine editing efficiencies has been shown to be comparably accurate to NGS techniques, and therefore is the recommended analysis tool for *in vivo* sgRNA validation using protoplasts.

Based on previous *in vivo* transformation studies, a sgRNA with a protoplast mutation rate of at least 10% is a viable candidate for recovering edited plants using other methods (Shan et al., 2014; Wang et al., 2016). Zhang et al. (2016) successfully transformed callus tissue of the variety Kenong199 via biolistic bombardment, using the same *GW2-B* sgRNA target as this study. They obtained mutagenesis frequencies of 2.3% with stable plasmid transformation, 2.6% with transient plasmid constructs, and 1.1% with *in vitro* synthesized transcripts. Zhang et al. (2019) used a different sgRNA targeting the same *GW2* gene to stably transform immature embryos of the variety Fielder at an average rate of 10% using *Agrobacterium*. The 19.2% and 36% editing efficiencies achieved in this study for *GW2-B* protoplasts is well above the 10% threshold, which reinforces the use of *GW2-B* as a positive control gene target, as well as shows the accuracy of this protocol for screening potential sgRNA candidates *in vivo* to be used for a multitude of downstream applications. The 16.4% and 12.9% editing efficiencies achieved for *ASN2-A* are also above this 10% threshold. Therefore, *ASN2-A* is a candidate for CRISPR-Cas9 transformation of wheat plants to reduce acrylamide formation during baking, which is an important trait for many food crops but does not yet exist in bread wheat germplasm. The consistent 0% editing efficiency for *PinB-D* demonstrates the utility of this protocol for screening sgRNAs *in vivo* before beginning full transformations in plants. A significant amount of time and resources can be saved by discovering at this stage that a sgRNA target is inaccessible, rather than after attempting transformation and regenerating multiple plants with no positive results.

## Conclusion

This research demonstrates the effectiveness of our simplified protocol for protoplast isolation from wheat leaf tissue, subsequent transformation with CRISPR-Cas9 RNPs and GFP plasmids, and analysis with online deconvolution software. While other protocols are available for protoplast isolation and transformation, there is a need for consensus and clarity in some steps. For example, vacuum infiltration of the enzyme solution was found to be unnecessary for isolation of sufficient, viable protoplasts despite being considered critical in the five studies by Shan et al. (2014), Wang et al. (2016), Liang et al. (2017, 2018), Cui et al. (2019). Researchers attempting to integrate *in vivo* testing of CRISPR sgRNA gene targets will also benefit from information presented in this study on expected viability of isolated protoplasts, total protoplast yield, and protoplast concentration for two common varieties. During the protoplast transformation steps, the other studies recommend only a single concentration and/or amount of cells, cell volume, plasmid/RNP amount, and plasmid/RNP volume. These recommendations are also different for each study. Concentrations and volumes of sgRNA and Cas9 can vary widely depending on the reagent manufacturer, modifications to the reagents, and *in vitro* synthesis yields, making exact specifications difficult to meet in every situation. The recommended ratios provided in this study will simplify this step for researchers with access to various reagents. Another aspect of CRISPR-Cas9 protoplast transformation that has been simplified in this study is the detection and analysis of editing in the cells. Wang et al. (2016) and Cui et al. (2019) analyzed protoplasts using NGS and HTS, which are accurate, but expensive, inaccessible to many researchers, time consuming, and comparatively difficult to analyze. Shan et al. (2014) and Liang et al. (2017, 2018) utilized PCR/RE assays to assess editing efficiency in protoplasts. This technique is relatively simple and inexpensive, but it can impede sgRNA design and does not provide details on types of indels or frequencies of those indels. In this study it was shown that Sanger sequencing followed by deconvolution analysis with ICE gave detailed editing efficiencies which were comparable to the other studies.

This study demonstrates the effectiveness of a simplified protocol for use in any lab attempting to isolate protoplasts and/or implement CRISPR transformation, which gives comparable results to the other available methods. This protocol requires less specialized equipment, provides easily replicable steps, reports more comprehensive results, and provides greater flexibility for other researchers attempting protoplast transformation and sgRNA validation, thus making implementation in any lab straightforward.

## Data Availability Statement

The raw data supporting the conclusions of this article will be made available by the authors, without undue reservation, to any qualified researcher.

## Author Contributions

KB, HG, NM, and RZ conceived the study. KB, HG, and NM designed the study and performed the experiments. KB and HG analyzed and interpreted the results. KB prepared the manuscript. HG, NM, and RZ edited the manuscript. All authors read and approved the manuscript.

## Conflict of Interest

The authors declare that the research was conducted in the absence of any commercial or financial relationships that could be construed as a potential conflict of interest.
